# Effects of Controlled Water Activity on Microbial Community Succession and Flavor Formation in Low-Salt Chili Mash Fermentation

**DOI:** 10.3390/foods15020360

**Published:** 2026-01-19

**Authors:** Linli Dai, Xin Wang, Nurul Hawa Ahmad, Jae-Hyung Mah, Wen Qin, Xinyao Wei, Shuxiang Liu

**Affiliations:** 1 College of Culinary and Food Science Engineering, Sichuan Tourism University, Chengdu 610100, China; 2 College of Food Science, Sichuan Agricultural University, Ya’an 625014, China; 3 Department of Food Science, Faculty of Food Science and Technology, Universiti Putra Malaysia, Serdang 43400, Malaysia; nurulhawa@upm.edu.my; 4 Department of Food and Biotechnology, Korea University, Sejong 30019, Republic of Korea; nextbio@korea.ac.kr; 5 College of Biological Science and Engineering, Fuzhou University, Fuzhou 350108, China; xwei@fzu.edu.cn

**Keywords:** low-salt chili mash, water activity, microbial diversity, volatile flavor substance

## Abstract

Although fermented seasonings play a pivotal role in improving food quality, the high sodium content of many traditional products poses considerable public health concerns, including hypertension and cardiovascular disease. This study established a low-salt fermentation strategy for Mumashan chili by regulating water activity (a_w_) under NaCl concentrations ranging from 4 to 12% (*w*/*w*). The a_w_-regulated system effectively maintained a_w_ within ± 0.03 at both 25 and 40 °C, thereby sustaining stable microbial activity despite the reduced salt concentration. Compared with the control group 15% NaCl, the 4% NaCl treatments exhibited significantly higher total acidity (130–200 g/kg vs. 24–58 g/kg) and a faster consumption rate of reducing sugars, with MH12 achieving an 80% rate of reducing sugars by day 21. Sensory evaluation revealed a higher overall quality score for the low-salt chili mash (MH12, 7.7/10), which was associated with a balanced aroma profile and enhanced color stability (ΔE < 5). However, the elevated relative abundance of opportunistic pathogens (*Klebsiella* app., ~10%) highlights the necessity of strict raw material hygiene. Overall, these results validate the feasibility of a_w_ regulation for low-salt fermentation, elucidate the associations between microbial communities and flavor development, and provide a basis for future industrial applications.

## 1. Introduction

Traditional high-salt fermented foods, such as chili bean paste, are widely produced as both artisanal and industrial products via spontaneous or controlled fermentation of chili mash. This process not only imparts characteristic savory, umami, and aromatic notes but also extends shelf life and improves safety through acidification and the competitive dominance of beneficial microorganisms, primarily lactic acid bacteria (LAB) [1]. Typically, these products contain 15–22 g of salt per 100 g of product. In conventional fermentation, salt fulfills multiple essential roles: it selectively inhibits spoilage and pathogenic bacteria, modulates microbial succession, facilitates texture formation, and extracts water and soluble compounds that shape the final flavor profile [2,3]. Excessive dietary salt intake is strongly associated with increased risks of hypertension, cardiovascular disease, osteoporosis, and kidney injury [4,5]. Although NaCl concentrations of 15–22% are traditionally employed to suppress spoilage microorganisms, reducing dietary salt intake is widely recognized as one of the most cost-effective strategies, and in some cases even cost-saving [6]. However, lowering salt levels in foods may adversely affect processing performance, textural properties, and preservative efficacy [7]. Ahmed et al. [8] reported that salt reduction decreased cheese cohesiveness and deformability, reduced dehydration shrinkage during ripening, and lowered hardness, resulting in a softer, more crumbly texture. Microbial interventions, such as starter cultures of *Pediococcus pentosaceus*, can enhance sensory qualities [9].

Current salt-reduction strategies for fermented chili products still have notable limitations. For low-salt chili fermentation facilitated by LAB and yeasts inoculation, the preparation and standardization of starter cultures remain challenging [10]. The selection, scale-up, and maintenance of viability and metabolic activity in commercial strains require considerable technical expertise, which may complicate practical implementation [11]. Moreover, pretreatments of chili raw materials aimed at reducing the initial microbial load may increase processing costs while preserving the quality attributes of the raw materials [12]. For instance, auxiliary methods such as ozone treatment and irradiation typically rely on specialized equipment, potentially raising capital investment and imposing a heavier financial burden on small- and medium-sized enterprises [13,14].

Notably, the fermentation of chili mash is dominated by a dynamic consortium of microorganisms. Key bacterial groups, predominantly LAB (*Lactobacillus* spp. and *Pediococcus* spp.), are crucial for accelerating fermentation, which stabilizes the system against pathogens colonization [15]. Concurrently, yeasts (e.g., *Zygosaccharomyces* spp., *Candida* spp.) and filamentous fungi contribute to enzymatic hydrolysis, alcohol biosynthesis, and the formation of characteristic esters and aromatic compounds [16]. This fermentation process is governed by microbial succession: the early dominance of acid-tolerant LAB establishes a stable acidic environment, followed by the proliferation of aroma-producing yeasts and fungi that determines the final flavor profile. While high salt concentrations traditionally suppress spoilage microorganisms, they constitute a crude approach that can also inhibit the growth and metabolic activity of these desirable functional microbes [17].

Water activity (a_w_) is defined as the ratio of the water vapor pressure above a food matrix to that above pure water, and it regulates microbial growth. Yeasts and molds can grow at a_w_ values of approximately 0.88 and 0.70, respectively [18,19]. The control of a_w_ is a well-established preservation strategy in various fermented foods. For instance, in fermented meats (e.g., dry-cured sausages) and cheeses (e.g., hard and semi-hard varieties), reducing a_w_ via drying or salting effectively restricts the growth of undesirable microorganisms while facilitating the development of characteristic textures and concentrated flavors [20]. Pre-drying to lower a_w_ can partially substitute the antimicrobial effect traditionally exerted by NaCl, enabling salt reduction without compromising microbial control, provided the target a_w_ and relevant microorganisms are considered [21]. Lower a_w_ conditions may favor acid-producing bacteria and aroma-active yeasts, which may accelerate fermentation and enhance flavor complexity [22]. However, the combined effects of a_w_ regulation and salt reduction on microbe–metabolite dynamics in chili mash remain insufficiently characterized.

Fermentation-derived volatiles—such as esters (e.g., ethyl hexadecanoate), pyrazines (e.g., tetramethylpyrazine), and alcohols (e.g., phenethyl alcohol)—shape the sensory identity of chili. These compounds are generated through microbial metabolism: *Kodamaea* spp. contribute to ester formation via enzymatic pathways, whereas *Staphylococcus* spp. modulates amino acid-derived aroma compounds [17]. Nevertheless, salt reduction can destabilize microbial consortia, promote the proliferation of opportunistic pathogens, and disrupt flavor balance. For instance, Guo et al. [23] reported elevated levels of three opportunistic pathogens—*Enterobacter* spp., *Pantoea* spp., and *Brevundimonas* spp.—in low-salt bean paste-meju, which could compromise product safety. Thus, a deeper understanding of a_w_-modulated microbial interactions is required to preserve flavor while ensuring safety in low-salt fermentation systems.

This study aimed to establish a synergistic a_w_–salt regulatory framework for low-salt chili mash fermentation. The overarching objective was to evaluate whether a_w_ modulation can reduce NaCl while maintaining product safety and flavor quality. Specifically, we aimed to (1) characterize physicochemical indices (total acidity and amino nitrogen) and microbial dynamics under controlled a_w_ conditions (0.87 and 0.91) at 25 °C with 4–12% NaCl, and (2) correlate microbial diversity with volatile profiles across different chili varieties.

## 2. Materials and Methods

### 2.1. Materials and Chemicals

Capsaicin and dihydrocapsaicin standards were purchased from TanMo Reference Materials Company Ltd. (Chengdu, China). Methanol and tetrahydrofuran were purchased from Shanghai Aladdin Biochemical Technology Co., Ltd. (Shanghai, China). NaCl (Table salt) was obtained from Sichuan Salt Industry Group Co., Ltd. (Chengdu, China). All other reagents used were of analytical grade.

### 2.2. Raw Materials and Fermentation Design

Chili peppers of the cultivar Mumashan Erjingtiao (*Capsicum annuum*) were purchased from Shuangliu District (Chengdu, China) as raw material. Fresh chilies were rinsed with water, hot-air-dried at 60 °C (a_w_ at 25 °C: 0.93), and then mixed with other components according to the formulation in Table 1 to prepare chili mash for fermentation. Five formulations were prepared: MH4, MH8, and MH12 (Mumashan Erjingtiao mash containing 4%, 8%, and 12% NaCl, respectively), and MX12 and MX15 (mixed-chili mash containing 12% and 15% NaCl, respectively). Each mash was transferred to 5 L jars sterilized by boiling-water blanching. The jar mouths were sealed with 5% brine, and fermentation was conducted out at 40 °C in a biochemical incubator (Shanghai Zhicheng Analytical Instrument Manufacturing Co., Ltd., Shanghai, China, ZXSD-R1270). During fermentation, samples were collected at 0, 7, 14, 21, 28, 35, and 45 d, placed into sterile sampling bags, and stored in a −20 °C freezer after air expulsion, awaiting subsequent analysis. MX12 and MX15 served as controls, with a_w_ values of 0.91 and 0.87 at 25 °C, respectively.

### 2.3. Physicochemical Analysis

The a_w_ of each chili sample was measured using an Aqualab 4TE meter (METER Group, Inc., Pullman, WA, USA) calibrated with saturated salt solutions at 25 and 40 °C. Moisture content was determined using a VM-E01 halogen moisture analyzer (Jiangsu Weikete Instrument Co., Taizhou, China). Total acidity and amino acid nitrogen were quantified in accordance with Chinese National Standards GB 12456-2021 [24] and GB 5009.235-2016 [25], respectively. Reducing sugars were determined via the 3,5-dinitrosalicylic acid (DNS) method with a glucose standard curve (Table S1). Absorbance was measured at 540 nm using a Varioskan Flash microplate reader (Thermo Fisher Scientific, Waltham, MA, USA). Capsaicinoids (capsaicin and dihydrocapsaicin) were quantified by high-performance liquid chromatography (HPLC, U3000, Thermo Fisher Scientific, MA, UAS) following Chinese National Standard GB/T 21266-2007 [26], with sample preparation performed via hot-air drying at 60 °C. Color parameters (L*, a*, and b*) were measured using a handheld colorimeter (CR-400, KONICA MINOLTA, Shanghai, China). Microbial counts (CFU/g) were determined by plate counting according to GB 4789.2-2022 [27]. The pH of chili mashes was measured using a PHS-3E pH meter (Shanghai Leici Instrument Co., Shanghai, China). Detailed procedures are provided in the Supplementary Materials.

### 2.4. Volatile Compound Profiling

Volatile compounds were extracted via headspace solid-phase microextraction (HS-SPME) using a divinylbenzene/carboxen/polydimethylsiloxane (DVB/CAR/PDMS) fiber [28]. A 3.000 g sample (±0.001 g) was weighted into a 20 mL amber headspace vial. Saturated NaCl solution (5 mL) was added, the vial was sealed, and the mixture was gently vortexed. The vial was equilibrated in a 55 °C water bath for 20 min to facilitate the partitioning of volatiles into the headspace. Volatiles were then adsorbed onto the DVB/CAR/PDMS fiber for 40 min. Subsequent analyses were performed using an Agilent 7890A–5975C GC–MS system equipped with a DB-5MS capillary column (30 m × 0.25 mm × 0.25 μm film thickness). The oven temperature program was set as follows: initial hold at 40 °C for 2 min, increased to 100 °C at 10 °C/min, then to 115 °C at 1 °C/min, followed by a ramp to 160 °C at 3 °C/min, and finally elevated to 250 °C at 10 °C/min (hold 5 min). Mass spectra were acquired in electron ionization mode at 70 eV over an *m*/*z* range of 40–450. Compounds were identified by matching mass spectra to the NIST14 library (match factor > 80%) and were semi-quantified via peak-area normalization.

### 2.5. Microbial Community Analysis

Total community genomic DNA was extracted using the E.Z.N.A.^®^ Soil DNA Kit (Omega Bio-teck, Norcross, GA, USA). DNA integrity was assessed by 1% agarose gel electrophoresis. DNA concentration and purity were determined using a NanoDrop 2000 spectrophotometer (Thermo Fisher Scientific). The V3–V4 region of the bacterial 16S rRNA gene was amplified using barcoded primers 338F (5′-ACTCCTACGGGAGGCAGCAG-3′) and 806R (5′-GGACTACHVGGGTWTCTAAT-3′). PCR was performed under the following conditions: initial denaturation at 95 °C for 3 min; 29 cycles of denaturation at 95 °C for 30 s, annealing at 53 °C for 30 s, and extension at 72 °C for 30 s; and a final extension at 72 °C for 10 min, followed by a hold at 4 °C. The fungal ITS1 region was amplified using barcoded primers ITS1F (5′-CTTGGTCATTTAGAGGAAGTAA-3′) and ITS2R (5′-GCTGCGTTCTTCATCGATGC-3′). PCR for the ITS1 region was conducted under the following conditions: initial denaturation at 95 °C for 3 min; 35 cycles of denaturation at 95 °C for 30 s, annealing at 55 °C for 30 s, and extension at 72 °C for 30 s; and a final extension at 72 °C for 10 min. Each 20 μL PCR reaction mixture contained 4 μL of 5 × TransStart FastPfu buffer, 2 μL of 2.5 mM dNTPs, 0.8 μL of each primer (5 μM), 0.4 μL TransStart FastPfu DNA polymerase, 10 ng template DNA, and nuclease-free water to reach the final volume. PCR amplicons were excised from 2% agarose gels, purified using a gel extraction/purification kit, and quantified with a Qubit 4.0 fluorometer. Sequencing libraries were constructed using the NEXTFLEX Rapid DNA-Seq Kit (Bioo Scientific, Austin, TX, USA) according to the manufacturer’s protocol, including adapter ligation, magnetic bead-based cleanup to remove adapter dimers, PCR enrichment, and final bead purification. Libraries were sequenced on an Illumina MiSeq platform (PE300; Shanghai Meiji Biomedical Technology Co., Ltd., Shanghai, China).

### 2.6. High-Throughput Sequencing Data Analysis

Paired-end raw reads were quality-filtered using fastp (v0.19.6) and merged using FLASH (v1.2.11) [29]. The preprocessing steps were as follows: (1) trimming low-quality bases (Q < 20) at read ends using a 50 bp sliding window; (2) merging paired reads based on overlapping (minimum overlap length: 10 bp); (3) discarding read pairs where the maximum mismatch ratio in the overlapping region exceeded 0.20; and (4) demultiplexing samples using barcodes and primers, with zero barcode mismatches allowed, up to two primer mismatches permitted, and reads oriented correctly.

Following quality filtering and merging, sequences were denoised using the DADA2 plugin in QIIME 2 with default parameters [30,31]. Denoised sequences were defined as amplicon sequence variants (ASVs). ASVs assigned to chloroplasts or mitochondria were removed to eliminate non-target sequences. To mitigate the impact of uneven sequencing depth on alpha-diversity estimates, all samples were rarefied to 20,000 sequences per sample, resulting in an average sequence coverage of 99.09% post-rarefaction. Taxonomic classification was conducted in QIIME 2 using a Naive Bayes classifier trained on the SILVA 16S rRNA gene database (v138). Functional profiles were predicted from 16S rRNA gene data using PICRUSt2 (v2.2.0) [32].

### 2.7. Sensory Evaluation

Sensory evaluation of samples fermented for 45 days was performed by ten trained student panelists using a 10-point hedonic scale (Table S2), with color weighted at 20% and aroma at 80%. Color assessment focused on hue (ranging from red to brown) and gloss, while aroma evaluation considered intensity, harmony, and the presence of off-odors. Scores were averaged across all replicates to ensure reliability.

### 2.8. Statistical Analysis

Prior to parametric analysis, the normality of the data distribution for each group was assessed using the Shapiro–Wilk test, and the homogeneity of variances was verified using Levene’s test. All key datasets met the assumptions of normality (*p* > 0.05) and homogeneity of variances (*p* > 0.05). One-way analysis of variance (ANOVA) followed by Tukey’s post hoc test was conducted using SPSS (v27.0.1, IBM Corp., Armonk, NY, USA), including total viable counts, the relative abundance of dominant bacterial phyla, and genera, as well as dominant fungal genera. Significance was set at *p* < 0.05. Principal component analysis (PCA) was performed using SIMCA-P (v16.0; Startorious Stedium Data Analytics, Umea, Sweden) to visualize temporal changes in volatile profiles. Orthogonal projections to latent structures–discriminant analysis (OPLS-DA) was conducted using SIMCA-P to identify volatiles that discriminated between the initial (day 0) and final (day 45) fermentation stages. The OPLS-DA model was validated using 200 permutation tests, and variables with a variable importance in projection (VIP) score > 1.0 were considered important. The microbial diversity indices (Sobs, Chao1, and Shannon) were calculated on an Illumina MiSeq platform (PE300; Shanghai Meiji Biomedical Technology Co., Ltd., Shanghai, China). The formula is as follows:

(1) *Chao*1 = *Sobs* + [*n*_1_(*n*_1_ − 1)/2(*n*_2_ + 1)]

In the formula, Sobs represents the actual number of observed species; n_1_ denotes the number of species observed only once; and n_2_ denotes the number of species observed only twice.

(2) *Shannon* = −Σ[*pi* × *ln*(*pi*)]

The pi denotes the proportion of sequences belonging to the ith species to the total number of sequences.

Additionally, Spearman’s rank correlation analysis was conducted via Origin (2024; OriginLab Corp., Northampton, MA, USA) to evaluate associations between the relative abundances of dominant genera and the concentrations of key volatile compounds. All experiments were performed in triplicate to ensure reproducibility.

## 3. Results and Discussion

### 3.1. Construction of Low-Salt Fermentation Systems

**Figure 1:**
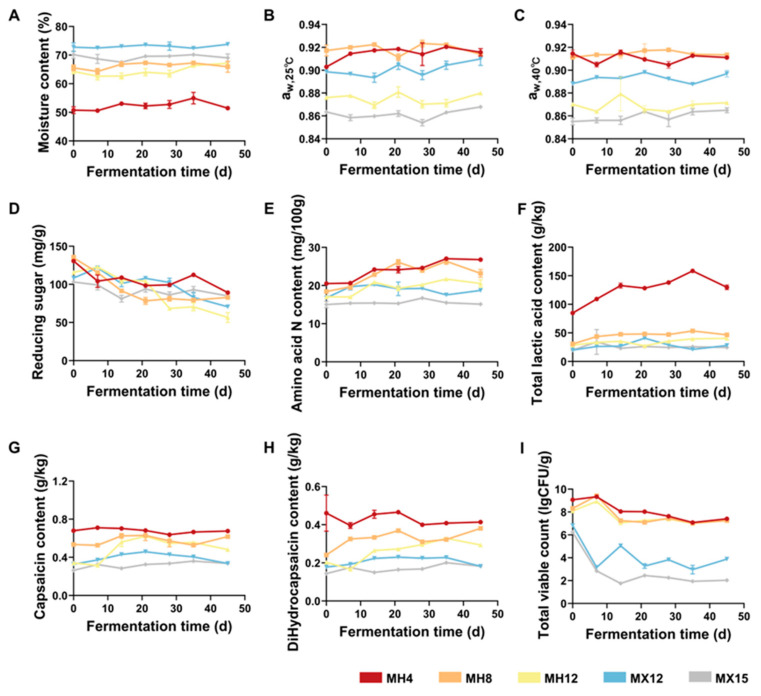
Physical and chemical indexes of different groups of chili mash fermentation during fermentation. (**A**) Change in water content. (**B**) aw of chili mash at 25 °C. (**C**) a_w_ of chili mash at 40 °C. (**D**) Change in reducing sugar content. (**E**) Changes in amino acid nitrogen content. (**F**) Change in total acid content. (**G**) Change in capsaicin content. (**H**) Change in dihydrocapsaicin content. (**I**) Changes in total viable bacterial count.

To accommodate the distinct a_w_ requirements of fermentative microorganisms, the NaCl levels were adjusted by modifying the proportion of dried chili peppers to achieve target a_w_ values at 25 °C (0.87 and 0.91, respectively). This adjustment established a controllable a_w_ regulation system to optimize the metabolic microenvironment for LAB and *Saccharomyces* spp. Moisture content was measured prior to fermentation, ranging from 50.7% to 73.7% across chili samples. The low-salt treatments (4% NaCl) exhibited significantly lower moisture content than the higher-salt treatment (12% NaCl; Figure 1A). Moisture content did not change significantly during fermentation, indicating stable process conditions with minimal environmental variability and facilitating consistent control across treatments. Subsequently, a_w_ was monitored to confirm that target values were maintained. As shown in Figure 1B, the a_w_ values of MH4 and MH8 remained within the setpoint of 0.91 ± 0.02 throughout fermentation at 25 °C. In MX12, a_w_ fluctuated around 0.90 ± 0.03, whereas MH12 and MX15 were maintained within 0.87 ± 0.02. Overall, a_w_ regulation (at 25 °C, 0.87–0.91) promoted stable fermentation at reduced salt levels (4–12% NaCl), as indicated by small a_w_ fluctuations (±0.02–0.03) at both 25 °C and 40 °C (*p* > 0.05; Figure 1B,C).

### 3.2. Effect of Low-Salt Systems on Chili Fermentation

During fermentation, microorganisms consume reducing sugars in the chili mash, converting them into various acids, esters, and other metabolites [33]. As shown in Figure 1D, the reducing sugar content generally decreased throughout the fermentation process. MH12 exhibited the most significant overall reduction in reducing sugars. Notably, reducing sugars declined sharply after day 21, indicating accelerated sugar consumption during this stage. MH4 showed the greatest reduction in reducing sugars from days 0–7, reflecting more rapid sugar utilization in the early fermentation stage. In contrast, MX15 displayed limited reducing sugar consumption, suggesting slower microbial growth or reduced metabolic activity under higher-salt conditions. The reducing sugar contents in MX12 also decreased significantly during fermentation. Collectively, these results demonstrate that salt level influences microbial utilization of reducing sugars during the fermentation of the same raw chili material. Compared with MX15, fermentation progressed more effectively in MH12, where MX15 only exhibited a small decrease in reducing sugars (18.27 mg/g). This difference may be attributed to the lower salt level in MH12 and the partial loss of capsaicinoids during 60 °C drying, which could reduce antimicrobial pressure and facilitate microbial activity [34].

Amino acid nitrogen was quantified using a calibration curve (Y = 0.04366X − 0.007104, R^2^ = 0.9968; Figure S1) in accordance with the national standard, indicating good linearity across the measured range. As shown in Figure 1E, amino acid nitrogen generally increased as fermentation progressed. During the early stage (days 0–7), amino acid nitrogen changed minimally (*p* > 0.05), consistent with an adaptation phase characterized by relatively low protease activity and limited protein hydrolysis by the microbial community. During the mid-fermentation stage (days 7–21), amino acid nitrogen increased significantly in all groups except MX15. During late fermentation (days 28–45), amino acid nitrogen did not change significantly in any groups. This plateau may reflect substrate limitation (i.e., depletion of fermentable proteins) and a pH decrease driven by lactic acid production from LAB, which can inhibit protease activity and limit further amino acid nitrogen formation. Notably, MX15 showed no significant change in amino acid nitrogen throughout fermentation, suggesting limited protein degradation and potentially weak fermentation activity under 40 °C incubation conditions.

Additionally, as shown in Figure 1F, the 4% NaCl treatment exhibited substantially higher total acidity than the higher-salt treatments, with MH4 reaching 130 g/kg. These results indicate that low-salt conditions enhance the activity of acid-producing microorganisms. However, excessively low salt levels may lead to over-acidification and an overly sour sensory profile. Therefore, under the tested a_w_ conditions, 4% NaCl was insufficient to restrain acid production by acidogenic microorganisms.

Furthermore, to further characterize fermentation across treatments, capsaicin and dihydrocapsaicin were quantified throughout the fermentation process. The calibration curves showed good linearity (capsaicin: Y = 0.1022X − 0.03156, R^2^ = 0.997; dihydrocapsaicin: Y = 0.1144X − 0.2232, R^2^ = 0.9932), supporting reliable quantification (Figure S2). Overall, capsaicinoid concentrations did not differ significantly among treatments during fermentation; however, a slight increase was observed in most groups from days 0–14 (Figure 1G,H). This early rise may be attributed to salt-induced disruption of chili tissues, which enhanced capsaicinoid release and increased the measured concentrations. In later fermentation stages, capsaicin showed a slight, non-significant decreasing trend in some groups, particularly under low-salt conditions. This pattern may reflect microbial degradation of capsaicinoids (e.g., by *Bacillus subtilis*), which appeared inefficient under the 40 °C incubation conditions used here [35].

To evaluate microbial proliferation under different salinity and a_w_ conditions, total viable counts were quantified throughout fermentation (Figure 1I). Statistical analysis of total viable counts across all treatment groups (MH4, MH8, MH12, MX12, MX15) confirmed a significant main effect of salt level (*p* < 0.01, One-Way ANOVA). Total viable counts decreased significantly with increasing salt content (*p* < 0.05, Tukey’s test), indicating that lower salt levels better supported microbial growth and activity. Notably, the Mumashan Erjingtiao mashes (MH4, MH8, MH12) exhibited viable counts 4–6 log units higher than those of MX12 during mid-to-late fermentation (days 14–45). This is consistent with the higher microbial diversity observed in the sequencing analysis (Table S3) and indicates more dynamic community succession. These viable-count data support the assessment of microbial stability in low-salt systems under a_w_ control.

**Figure 2:**
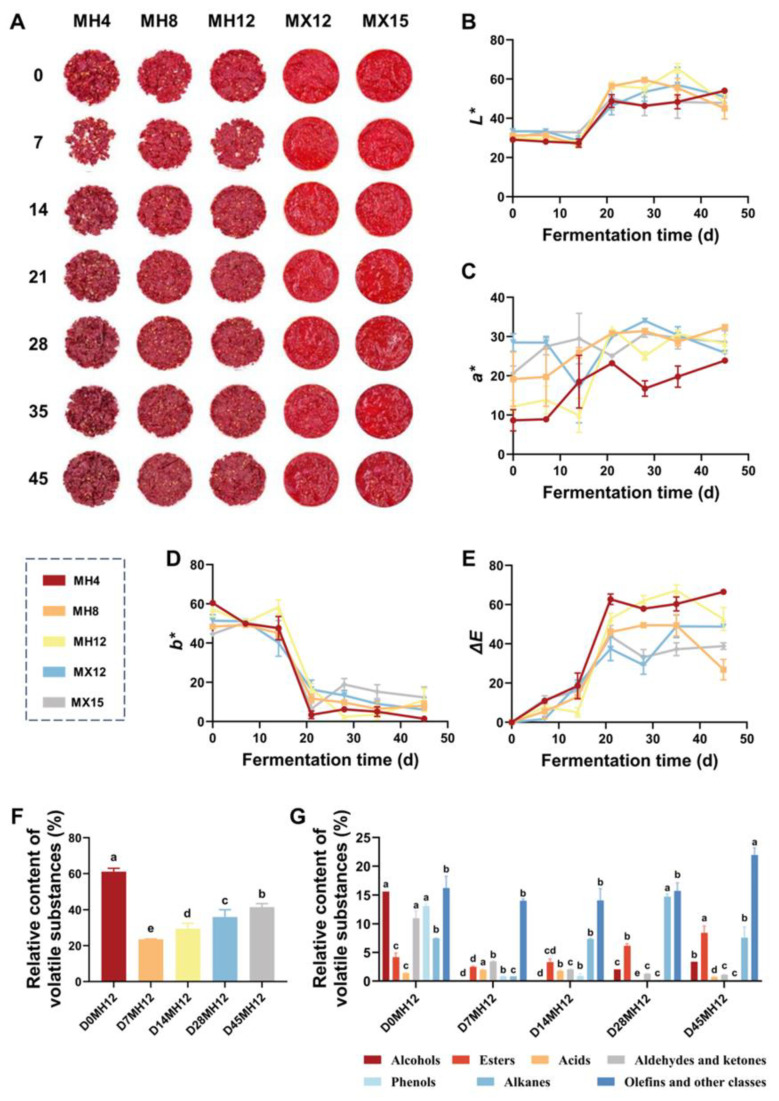
(**A**) Chili mash fermentation process. (**B**) L*-value. (**C**) a*-value. (**D**) b*-value. (**E**) ΔE-value. (**F**) Relative contents of total volatile substances in MH12 of chili mash. (**G**) Relative contents of various volatile substances in MH12 of chili mash. The lowercase letters (a, b, c, d, e) represent significance markers from one-way analysis of variance followed by Tukey’s multiple comparison test (*p* < 0.05). Groups labeled with the same letter show no significant difference, while those with different letters indicate significant differences.

With respect to color stability during fermentation, the mixed-chili treatments exhibited significantly browner coloration than the fresh-chili treatments (Figure 2A). This difference likely stems from prior hot-air drying at 60 °C, which can promote thermal pigment degradation and facilitate Maillard reactions between sugars and amino acids, yielding brown-colored products that reduce perceived redness [36]. In the fresh-chili treatments, color changes were minimal during early fermentation (days 0–14), although ΔE values indicated perceptible differences (Figure 2B–E). This variability may be partly attributable to the uneven distribution of yellow seeds, which can interfere with colorimetric measurements. Overall, the limited color shift in the fresh-chili treatments suggests that salt may contribute to preserving chili color during fermentation.

Consistent with this trend, traditional chili mash typically shifts from bright red to reddish-brown or brown during prolonged fermentation, largely due to the degradation of capsaicinoids and other pigments [37]. Accordingly, aroma and color were weighted 8:2 ratio in the sensory evaluation to better capture changes driven by the accumulation of flavor-related metabolites during fermentation. As shown in Table 2, MH12 achieved the highest overall sensory score (7.70), whereas MX15 received the lowest (3.38). These sensory outcomes were consistent with differences in the raw-material physicochemical properties and the extent of metabolite accumulation during fermentation, supporting the appropriateness of the selected weightings.

### 3.3. Testing of Microbial Community and Fermentation Flavor During Low-Salt Fermentation

To characterize aroma development in the low-salt sample MH12, changes in volatile compounds were monitored throughout fermentation. As shown in Table S4, 51 volatile compounds were identified in MH12 at five time points (days 0, 7, 14, 28, and 45), including 7 alcohols, 8 esters, 2 acids, 7 aldehydes/ketones, 5 phenolics, 16 alkanes, and 6 terpenes/others. These compounds are major contributors to the chili mash aroma. As shown in Figure 2F, the total relative abundance of volatiles in MH12 decreased initially and then increased. The highest value occurred on day 0 (≈61%). By day 7, the total relative abundance decreased significantly (*p* < 0.05) to ~25%, and then gradually increased to ~43% as fermentation proceeded. On day 0, MH12 was characterized by relatively high proportions of alcohols (15.58%), aldehydes/ketones (10.94%), phenolics (13.10%), alkanes (7.47%), and olefins/other compounds (16.22%), reflecting the abundance of intrinsic volatiles in raw chili. The sharp decline on day 7 (from 61.11% to 23.59%) may reflect volatilization losses and microbial degradation or utilization of endogenous chili components during early fermentation. Thereafter, the total volatile abundance increased, reaching 41.52% by day 45. This trend aligns with the sensory evaluation results, as the gradual accumulation of volatiles in mid-to-late fermentation likely contributes to the balanced aroma profile of MH12.

As shown in Figure 2G, ester abundance in MH12 declined from days 0–7 and then increased, reaching 8.4% by the end of fermentation. Major esters included ethyl hexadecanoate (waxy, slightly sweet), ethyl dodecanoate (strong fruity notes), ethyl tridecanoate (waxy, slightly sweet), ethyl tetradecanoate (fruity), ethyl pentalate (fruity), ethyl undecanoate (fresh fruity, with sweet and floral notes), and methyl salicylate (sweet, minty). These esters are key contributors to the fruity and sweet aromatic notes of fermented chili mash. Aldehydes and ketones were present at high levels in unfermented samples (15.58%) but decreased significantly to ~3% during fermentation (*p* < 0.05), which may be attributed to microbial degradation or reduction reactions. Phenolics were initially detected at 13.10% but became undetectable by day 45, indicating complete utilization or degradation of these compounds by the microbial community. Alkane abundance fluctuated throughout fermentation, peaking at 14.73% on day 2. Dominant alkanes included 2-methyleicosane, 2-methyltridecane, and hexadecane, which exhibit limited odor activity and are typically described as weak or waxy, thus exerting minimal influence on the overall aroma profile. Olefins and other compounds constituted a substantial fraction throughout fermentation (>14%) and increased to ~22% by day 45. Notably, tetramethylpyrazine accounted for ~17.5% of the volatile fraction at the end of fermentation. This compound is commonly detected in fermented and roasted foods, contributing characteristic nutty and cocoa-like notes [38], which likely enhanced the flavor complexity of MH12 and contributed to its high sensory score.

**Figure 3:**
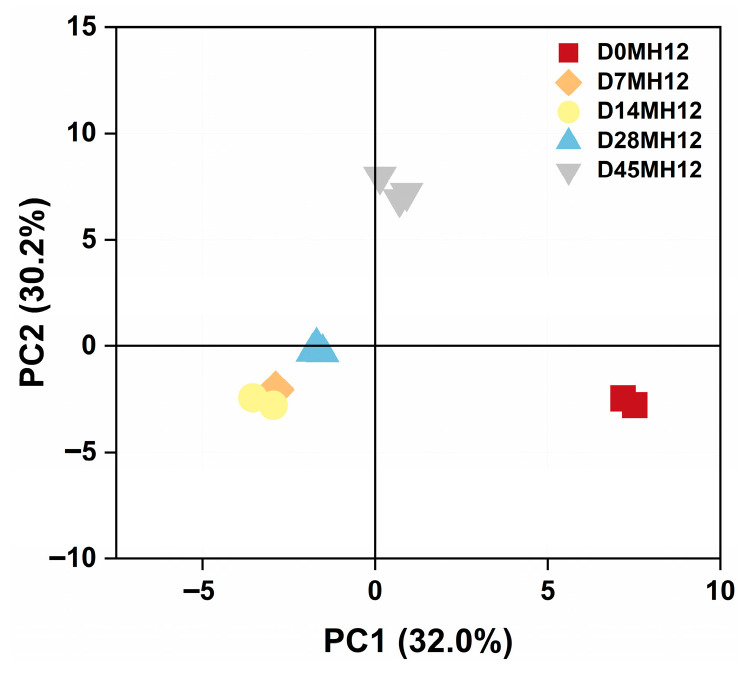
Principal component analysis of MH12 in chili mash.

To further visualize the temporal dynamics of volatile and characterize overall flavor changes during fermentation, principal component analysis (PCA) was performed on the volatile of MH12. As shown in Figure 3, samples from different fermentation stages separated clearly in the PCA space, with no obvious outliers. Replicates at each time point formed distinct clusters, indicating good reproducibility of the volatile data. Day 0 samples (D0MH12, red) clustered closely on the right side of the score plot, reflecting similar intrinsic volatile compositions in the raw chili material at the start of fermentation. During fermentation, some day 7 samples (D7MH12, orange) were located near day 14 samples (D14MH12, yellow), suggesting partial continuity in volatile profiles between these early-to-mid fermentation stages. Day 14 samples clustered mainly in the lower-left quadrant, showing clear separation from day 0 samples. This distinct partitioning implies that fermentation-associated biochemical processes, including microbial metabolism, substantially altered the volatile profile. Day 28 samples (D28MH12, light blue) were predominantly distributed in the mid-left region and were separated from other time points, indicating a unique volatile signature at this stage. Day 45 samples (D45MH12, gray) clustered in the upper region and were distinct from all earlier stages, suggesting that the volatile profile had evolved toward a characteristic end-point composition consistent with the mature flavor of MH12.

Building on the viable-count results and the flavor maturation observed in MH12, microbial community succession was further characterized by high-throughput DNA sequencing. Alpha diversity (within-sample diversity) was evaluated using Coverage, Sobs, Chao1, and Shannon indices (Table S3). Coverage exceeded 0.99 for all samples, and Chao1 values were comparable to Sobs values, indicating sufficient sequencing depth and reliable diversity estimates. After removing ASVs assigned to chloroplasts or mitochondria, taxa with a relative abundance < 0.01% were categorized as “Others”. Thereafter, although total viable counts stabilized or declined gradually during mid-to-late fermentation, the Shannon index remained high (2.8–3.0; Table S3), indicating that community richness and evenness were maintained. This stability in microbial diversity likely underlies the consistent flavor development observed in MH12, aligning with its high sensory score.

However, potential food safety risks were identified, as shown in Figure S3: the relative abundance of conditionally pathogenic genera, including *Enterobacter* and *Klebsiella*, increased during fermentation. This suggests that the resident fermentative microbiota did not sufficiently suppress their growth under the tested reduced-salt conditions (12% NaCl). This trend may be associated with a higher initial microbial load in the raw materials. In this batch, chilies were purchased from a local market where potential cross-contamination sources existed; this may have introduced opportunistic pathogens despite pre-mash preparation washing. These observations highlight raw-material hygiene as a critical control point for fermented chili mash production. If contamination occurs, microbial antagonism during fermentation may be insufficient to eliminate potential food safety hazards [39].

**Figure 4:**
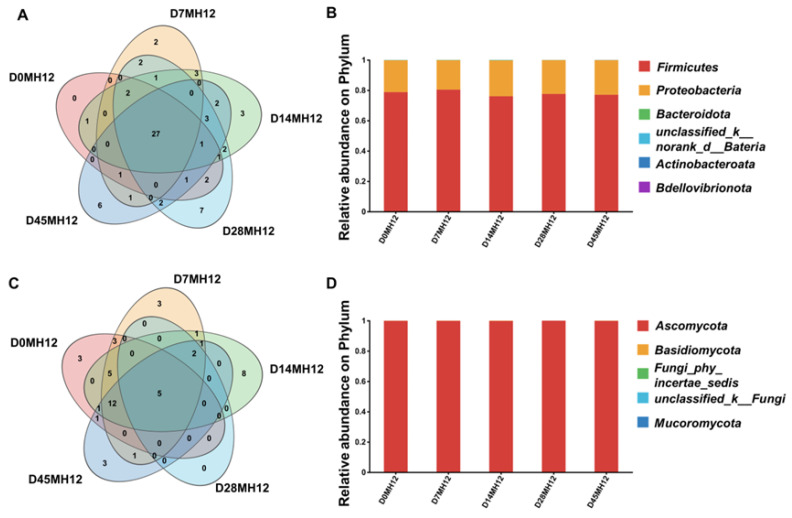
Determination of microorganisms of MH12 during fermentation. (**A**) The level Venn diagram of MH12 bacteria in chili mash. (**B**) Relative abundances of phylum of MH12 bacteria. (**C**) Horizontal Wayne diagram of MH12 fungi in chili mash. (**D**) Relative abundance of phylum in MH12 fungi in chili mash.

Fungi also play a pivotal role in flavor development during fermentation. As shown in Table S5, Chao1 values were comparable to Sobs values, and Coverage was 1.00, indicating sufficient sequencing depth and reliable fungal diversity estimates for MH12. The fungal Shannon index increased from 0.55 to approximately 1.0 throughout fermentation, suggesting a gradual increase in within-sample diversity. Notably, the fungal Shannon diversity was lower than bacterial Shannon diversity (Table S5), indicating that fungal communities were less diverse than bacterial communities in MH12. Figure 4C illustrates five predominant fungal taxa across fermentation: *Kodamaea*, *Pichia*, *Stagonosporopsis*, *Wallemia*, and *Dipodascaceae*, which represent key fungal members of the fermentation system. At the phylum level, Ascomycota dominated nearly the entire fungal community (99.99%; Figure 4D). At the genus level, the community was dominated by *Pichia*, *Kodamaea*, and *Zygosaccharomyces*, which together accounted for nearly 99% of the total sequences. From days 0–7, *Pichia* was dominant genus, representing ~95% of the genus-level relative abundance. Between days 7 and 28, the relative abundance of *Kodamaea* decreased progressively, reaching a minimum of 7.1% on day 28. During late fermentation (days 28–45), the relative abundance of *Pichia* increased to 36.8%. In contrast, *Zygosaccharomyces* was present at low levels initially but increased gradually toward the end of fermentation. Collectively, these results demonstrate alternating dominance among *Pichia*, *Kodamaea*, and *Zygosaccharomyces* during fermentation, which may contribute to the sweet and fruity aroma characteristics of MH12—consistent with the ester and tetramethylpyrazine accumulation observed in earlier volatile profiles. These dynamics likely reflect stage-dependent changes in environmental conditions (e.g., pH and nutrient availability) and competitive interactions among microorganisms [40,41].

To further dissect the links between microbial communities flavor formation, we examined associations between microbial abundance and volatile compounds, which can aid in identifying taxa contributing to flavor development and supporting targeted control of sensory characteristics in fermented foods [42]. Accordingly, orthogonal projections to latent structures–discriminant analysis (OPLS-DA) was used to compare volatile profiles of MH12 on days 0 and 45. Differential volatiles compounds were identified using variable importance in projection (VIP) score > 1 and *p* < 0.05. The OPLS-DA model exhibited strong performance (R^2^X = 0.804, R^2^Y = 0.993, Q^2^ = 0.985), with all values exceeding 0.5, demonstrating good explanatory and predictive ability. A 200-permutation test yielded R^2^ and Q^2^ intercepts on the negative axis, confirming the absence of model overfitting (Figure S4). Ten differential volatile compounds were identified via OPLS-DA (Table 3). To screen for core microbiota relevant to flavor, taxa with a relative abundance < 0.1% were excluded. The remaining taxa putatively associated with fermentation were retained for correlation analysis with volatile compounds. In MH12, 10 bacterial genera and 5 fungal genera met these criteria: *Bacillus*, *Weissella*, *Enterobacter*, *Lactococcus*, *Lactobacillus*, *Leuconostoc*, *Pediococcus*, *Acetobacter*, *Vagococcus*, and *Gluconobacter* (bacteria); and *Kodamaea*, *Pichia*, *Zygosaccharomyces*, *Aspergillus*, and *Candida* (fungi).

**Figure 5:**
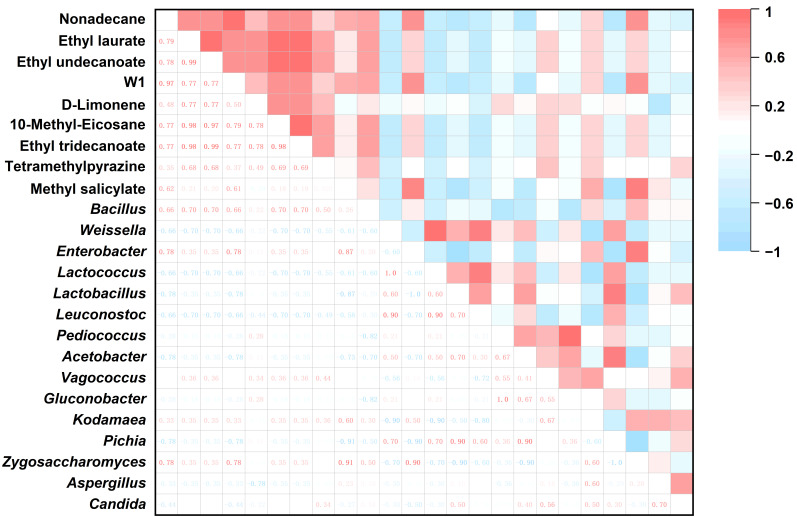
MH12 heat map of key volatile substances and flora in fermented chili mash.

Spearman’s rank correlation analysis was conducted to explore the associations between these core taxa (putatively linked to fermentation) and differential volatile compounds, as illustrated in Figure 5 and summarized in Table 3. For instance, n-nonadecane in MH12 exhibited a significant positive correlation with *Bacillus*, *Enterobacter*, and *Zygosaccharomyces*, alongside a positive correlation with *Kodamaea*. Ethyl dodecanoate showed significant positive correlations with *Bacillus* and positive correlations with *Enterobacter*, *Vagococcus*, *Kodamaea*, and *Zygosaccharomyces*. 2-Butyl-1,1,3-trimethylcyclohexane was significantly positively correlated with *Enterobacter*, *Zygosaccharomyces*, and *Bacillus*, with an additional positive correlation with *Kodamaea*. D-limonene correlated positively with *Bacillus*, *Enterobacter*, *Pediococcus*, *Acetobacter*, *Vagococcus*, *Gluconobacter*, and *Pichia*. 10-Methyl-20-octane and ethyl 13-caprylate both had significantly positive correlations with *Bacillus*, and positive correlations with *Enterobacter*, *Vagococcus*, *Kodamaea*, and *Zygosaccharomyces.* Tetramethylpyrazine correlated positively with *Bacillus*, *Enterobacter*, *Vagococcus*, *Kodamaea*, *Zygosaccharomyces*, and *Candida*. Methyl salicylate showed significantly positive correlations with *Enterobacter*, *Kodamaea*, and *Zygosaccharomyces*, as well as positive correlations with *Bacillus* and *Vagococcus* (Table 3). These associations validate the putative links between core microbiota and flavor formation, aiding in the identification of candidate taxa for targeted control of flavor development in low-salt chili fermentation. Guo et al. [23] reported similar microbial-flavor compound correlations in the low-salt broad bean paste-meju fermentation, particularly emphasizing the role of *Bacillus* and *Enterobacter* in flavor modulation. This consistency suggests these microorganisms may enhance flavors complexity via conserved metabolic pathways. In contrast, Zheng et al. [20] did not observe significant correlations between microbes and flavor compounds (e.g., pyrazines) in low-salt sausage fermentation, likely due to differences in the microbial community composition and fermentation conditions between food systems.

Collectively, these findings demonstrate that by regulating a_w_, we were able to maintain microbial stability and the activity of beneficial microorganisms, even at reduced salt concentrations (4–12% NaCl). These microorganisms not only contribute to rapid fermentation by enhancing acidity but also play a pivotal role in flavor development-particularly in the production of ester and pyrazine compounds, which align with the volatile profiles observed in MH12. Compared to traditional high-salt fermentation systems, a_w_ regulation enables salt reduction while preserving fermentation efficacy and flavor quality. Our results confirm that even with lower salt concentrations, appropriate a_w_ control can enhance acidity, improve flavor complexity (e.g., via tetramethylpyrazine and ester accumulation), and maintain color stability (ΔE < 5), establishing it as a feasible strategy for industrial low-salt chili mash production.

Mechanistically, this study successfully established an active microbial consortium dominated by *Bacillus* and key yeasts in low-salt chili mash through a_w_ regulation. The underlying mechanism lies in the fact that a moderate reduction in a_w_ (0.87–0.91) creates a selective pressure distinct from that of high salt concentration. This pressure more effectively inhibits most spoilage microorganisms with high water requirements, while many *Bacillus* and yeasts thrive and become dominant due to their robust osmoregulation systems [43]. This explains why the low-salt, a_w_-controlled system exhibited higher microbial diversity and more vigorous metabolic activity (e.g., acid production, reducing sugar consumption) than traditional high-salt systems, which are typically dominated only by extreme halophiles or tolerant species [44].

It is particularly noteworthy that *Bacillus* in our system showed a significant correlation with the accumulation of tetramethylpyrazine. This is likely because salt reduction alleviated the inhibitory effect on *Bacillus*—a contrast to high-salt environments that typically favor extreme halophiles or halotolerant species. With reduced salt stress, *Bacillus* can contribute the characteristic nutty flavor via metabolic pathways such as the Strecker degradation of amino acids [45]. This differs from the flavor formation mechanisms in some high-salt fermented bean pastes, highlighting the potential of low-salt fermentation to develop unique flavor profiles.

However, this study also objectively exposed the limitation of relying exclusively on a_w_ modulation: some drying-tolerant opportunistic pathogens (e.g., *Klebsiella*) may still survive and proliferate. This strongly suggests that future low-salt fermentation practices must adopt an integrated “hurdle technology” approach [46]. Based on a_w_ control, it is essential to strengthen strict microbiological quality control of raw materials and consider inoculating competitive starter cultures to establish a more robust microbial safety barrier. This will help translate the “feasibility” demonstrated in this study into industrial “reliability”.

## 4. Conclusions

This study presents a low-salt fermentation strategy for chili mash based on the precise regulation of a_w_, establishing its feasibility as an alternative to conventional high-salt preservation. Using Mumashan Erjingtiao chili, fermentation under controlled a_w_ (0.87–0.91) with reduced NaCl (4–12%) maintained process stability while retaining flavor complexity. The a_w_ regulation stabilized the fermentation process, supporting the dominance of LAB and yeasts under reduced-salt conditions. Shifts in microbial composition were linked to flavor differentiation. In MH12, *Bacillus* and dominant yeasts correlated with the accumulation of ester and pyrazines. The sensory quality of MH12 was superior to that of the high-salt control (7.70/10 vs. 3.38/10), consistent with improved color retention (ΔE < 5) and a more balanced aroma profile. However, opportunistic pathogens (e.g., *Klebsiella*) increased under reduced-salt conditions (up to 10% relative abundance), emphasizing raw-material hygiene as a critical control point. These findings support efforts to reduce dietary sodium by demonstrating that salt reduction can be achieved without substantial loss of sensory quality when a_w_ is appropriately controlled. The observed microbe–flavor relationships (e.g., associations between *Bacillus* and pyrazines) offer a foundation for targeted starter culture selection and process optimization in low-salt fermentations.

## Figures and Tables

**Table 1:** Controlled a_w_ chili mash formulations.

Name	Air Dried Chili Mash (g, a_w,25°C_ = 0.93)	Fresh Chili Mash (g, a_w,25°C_ = 0.99)	a_w,25°C_ of Mixed Chili Mash
4%Nacl/MH4	100	22.22	0.91
8%Nacl/MH8	100	200	0.91
12%Nacl/MH12	100	220	0.87
12%Nacl/MX12	—	100	0.9
15%Nacl/MX15	—	100	0.87

Note: “—” means no data.

**Table 2:** Controlled water activity chili formula.

Sample	MH4	MH8	MH12	MX12	MX15
score	5	5.24	7.7	4.24	3.38

**Table 3:** Key differential volatile compounds identified in MH12.

Sample	Day 0, MH12 (D0MH12, %)	Day 45, MH12 (D45MH12, %)	VIP
Positive 19 alkane	-	0.450 ± 0.023	1.11
Ethyl dodecanoate	-	0.630 ± 0.072	1.11
Ethyl 11 acid	-	0.440 ± 0.013	1.11
W1	-	0.650 ± 0.004	1.11
D-limonene	0.130 ± 0.004	0.250 ± 0.003	1.11
10-Methyl-eicosane	-	0.740 ± 0.410	1.11
Ethyl tridecaate	-	1.440 ± 0.128	1.10
Tetramethyl-pyrazine	10.150 ± 1.118	17.510 ± 1.88	1.03
Methyl salicylate	2.400 ± 0.208	3.440 ± 0.378	1.02

Note: “-” means no data; W1 stands for 2-butyl-1, 1, 3-trimethylcyclohexane.

## Data Availability

The original contributions presented in this study are included in the article/Supplementary Materials. Further inquiries can be directed to the corresponding author.

## Supplementary Materials

The following supporting information can be downloaded at: https://www.mdpi.com/article/10.3390/foods15020360/s1. Table S1. Glucose standard curve preparation. Table S2. Sensory evaluation table. Table S3. Index of bacterial Alpha diversity. Table S4. Relative content of MH12 volatile substances. Table S5. Index of Alpha diversity of fungi. Figure S1. Amino acid nitrogen standard curve. Figure S2. A. Standard curves of capsaicin and B. dihydrocapsaicin. Figure S3. Relative abundances of genus of MH12. Figure S4. The OPLS-DA models of D0MH12 and D45MH12 in chili mash were fitted with the result plots of 200 permutations.
