# Supplementary material for: Effects of Controlled Water Activity on Microbial Community Succession and Flavor Formation in Low-Salt Chili Mash Fermentation
Source: Foods. 2026 Jan 19;15(2):360. doi: 10.3390/foods15020360 (PMC12840632; doi:10.3390/foods15020360)
Supplement: Supplementary file 1 [file foods-15-00360-s001.zip › foods-4090832-supplementary.pdf]

Effects of Controlled Water Activity on Microbial Community Succession and Flavor Formation in Low-Salt Chili Mash Fermentation

Linli Dai[12, #], Xin Wang[2, #], Nurul Hawa Ahmad[3], Jae-Hyung Mah[4], Wen Qin[2], Xinyao Wei[5], Shuxiang Liu[2, *]

[1]Sichuan Tourism University, Chengdu Sichuan, 610100, China

[2]College of Food Science, Sichuan Agricultural University, Ya'an, Sichuan, 625014, China

[3]Department of Food Science, Universiti Putra Malaysia, Subang Jaya Selangor, Malaysia

[4]Department of Food and Biotechnology, Korea University, Sejong, Republic of Korea

[5]College of Biological Science and Engineering, Fuzhou University, Fujian, China

[*] **Corresponding Author**

Shuxiang Liu, sliu@sicau.edu.cn

No.46 Xinkang Road, Dongcheng Street, Yucheng District, Ya'an City, Sichuan Province, China.

[#] The authors contributed equally to the work.

1. Supplemental details of the methods

1.1 Moisture content determination

Weigh about 5.00 g of sample, measure the moisture content of the sample using a rapid moisture analyzer, and repeat the measurement 3 times.

1.2 Water activity (a_w) determination

When the measurement temperature is adjusted to 25°C, the temperature is adjusted to 25°C and 40°C respectively when the deviation from the measured value of the standard solution is not more than 0.001. Weigh about 3.00 g of the sample and put it into the a_w analyzer and repeat the measurement 3 times.

1.3 Total acidity

Weigh 25.00 g of the sample and place it in a 150 mL condenser conical flask, add 50 mL of CO_2-free water for 15 minutes, mix well, boil for 30 minutes, shake 2 - 3 times during the boiling water bath to allow the organic acids to fully dissolve in the water. After it cools, filter it with quick-cooking filter paper and collect the filtrate to be tested.

1.4 Determination of amino acid nitrogen content

Weigh 1.00 g of the sample, add it to a 100 mL volumetric flask, dilute it with ultrapure water to the tick line, mix well, and test.

1.5 Determination of reducing sugar content

Weigh 3.00 g of sample into a grinder, grind with 3 mL of distilled water, transfer to a 50 mL centrifuge tube, rinse the grinder with 15 mL of distilled water 2 - 3 times, collect the rinse and add it to the centrifuge tube. Place the 50 mL centrifuge tube in a 50°C water bath for 30 min. After cooling, centrifuge at 4000 g centrifugal force for 5 min at 4°C. Set aside the supernatant, add 20 mL of distilled water to the pellet and mix well, centrifuge at 4°C, 4000 g centrifugal force for 5 minutes. Collect the supernatant, add the supernatant to a 100 mL volumetric flask twice, add water to the scale to set the volume, to be

tested.

To make glucose standard curve: Take a 10 mL colorimetric tube and add the reagent according to Table S1, tube No. 1 is blank, detect the absorbance at 540 nm, take the absorbance value as the ordinate coordinate, and the standard solution concentration is the abscissa to make a standard curve.

Take 1 mL of the extract, add 2 mL of DNS reagent, and follow the same steps as the preparation standard curve, and measure the absorbance value at 540 nm after processing. The reducing sugar content is calculated according to Equation 1:

$$X=\frac{C\times V_1}{m\times V_2} \quad (1)$$

X: Reducing sugar content in the sample mg/mL; V_1: Total volume of extract solution, mL; m: mass of sample, g; V_2: Sample volume used in mL for assay.

1.6 Capsaicin content determination

Weigh about 10.00 g of sample, dry it in a hot air circulation oven at 60°C until the constant weight to obtain the sample to be tested.

1.7 Colorimetric determination

Weigh 20 - 30 g of chili samples in a sterile Petri dish, then measure 3 different positions with a handheld colorimeter to obtain a*, b*, and L* values 3 times. The unfermented chili liquor from day 0 of each group was used as the reference color of this group. The total color ΔE during the fermentation process is calculated according to Equation 2:

$$\Delta E=\sqrt{(\Delta L^*)^2+(\Delta a^*)^2+(\Delta b^*)^2} \quad (2)$$

1.8 Determination of total bacterial count

Weigh 25.00 g of the sample into a sterile sampling bag with 225 mL of sterile phosphate buffer solution and gradient dilution assay.

2. Supplemental table

Table S1 Glucose standard curve preparation

Material (mL)	1	2	3	4	5	6
Glucose standard solution (1 mg/mL)	0	0.2	0.4	0.6	0.8	1.0
Distilled water	1.0	0.8	0.6	0.4	0.2	0
DNS	2.0	2.0	2.0	2.0	2.0	2.0
Boiling water bath for 5 minutes, immediately remove and cool with tap water to room temperature						
Distilled water	9.0	9.0	9.0	9.0	9.0	9.0
Equivalent to the amount of glucose	0	0.2	0.4	0.6	0.8	1.0

Table S2 Sensory evaluation table

Factor	Judging criteria	Fraction
Color (10 points, 20%)	Bright red, shiny	8 - 10
	Dark red, shiny	6 - 7
	Reddish-brown, shiny	4 - 5
	Brown, dull	0 - 3
Aroma (10 points, 80%)	Fragrance is stronger, flavor is more harmonious, no peculiar smell	8 - 10
	Aroma is average, flavor is incongruous, no peculiar smell	5 - 7
	Poor odor coordination, no peculiar smell	3 - 4
	Strong odor	0 - 2

Table S3 index of bacterial Alpha diversity

Sample	Sobs	Chao 1	Shannon	Coverage
D0MH12	142	183.352941	3.009581	0.993298
D7MH12	126	151.375	2.910939	0.994885
D14MH12	129	146.4	3.04036	0.994709
D28MH12	119	131	2.918271	0.995767
D45MH12	112	148.25	2.895452	0.994709

Table S4 Relative content of MH12 volatile substances

Material	Relative content (%)				
	D0MH12	D7MH12	D14MH12	D28MH12	D45MH12
	Alcohols				
1-Hexanol	0.031 ± 0.0299	-	-	-	-
Cis-α, α-5-trimethyl-5-vinyltetrahydrofuran-2-methanol	-	-	-	0.441 ± 0.0445	-
2-Furanmethanol	-	-	-	-	0.384 ± 0.2047
Benzyl alcohol	-	-	-	-	0.585 ± 0.3808
Phenylethanol	4.167 ± 0.0521	-	-	1.910 ± 1.2228	2.571 ± 1.1256
Trans-linalool oxides (furans)	-	-	0.005 ± 0.0002		
Linalool	11.398 ± 0.0502	-	-	-	-
	Ester				
2-(5-methyl-5-vinyltetrahydrofuran-2-yl) propyl-2-ylcarbonate	-	-	-	0.706 ± 0.0135	-
Ethyl dodecanoate	-	-	-	-	0.627 ± 0.0717

Ethyl cetacanoate	1.288 ± 0.9551	-	-	0.787 ± 0.0438	1.526 ± 0.3459
Ethyl tridecaate	-	-	-	-	1.436 ± 0.1275
Ethyl tetrapanakylate	0.505 ± 0.0672	-	-	0.312 ± 0.0165	0.541 ± 0.0952
Ethyl pentaalkanate	-	-	-	0.219 ± 0.0139	0.396 ± 0.3435
Ethyl 11 acid	-	-	-	-	0.441 ± 0.0133
Methyl salicylate	2.396 ± 0.2077	2.493 ± 0.0773	3.298 ± 0.5454	4.123 ± 0.3225	3.442 ± 0.3776
Acids					
2-(3-oxobenzo [D] isothiazole-2 (3H)-yl) acetic acid	-	-	0.112 ± 0.0006	-	-
Acetic acid	1.396 ± 0.0505	1.974 ± 0.0602	1.690 ± 0.1201	-	0.730 ± 0.0735
Aldehydes and ketones					
(E)-2-Hexenal	2.042 ± 0.1005	-	-	-	-
2-Hexenal	2.482 ± 0.0472	-	-	-	-
Hexanal	0.613 ± 0.0951	-	-	-	-
Benzaldehyde	1.687 ± 0.7043	-	0.809 ± 0.0486	1.273 ± 0.0325	0.687 ± 0.0313

Hyacinthin	0.470 ± 0.0700	-	-	-	0.417 ± 0.0116
3-Hydroxy-2-butanone	3.655 ± 0.9003	3.433 ± 0.0608	-	-	-
1-(2-hydroxy-5-methylphenyl)-ethylketones	-	-	1.216 ± 0.0196	-	-
Phenols					
2,4-Di-tert-butylphenol	-	0.259 ± 0.0034	-	-	-
2-Methoxy-4-vinylphenol	-	0.565 ± 0.0090	0.889 ± 0.4093	-	-
2,5-Bis (1,1-dimethyl)-phenol	-	-	-	0.224 ± 0.0108	-
4-Ethyl-2-methoxyphenol	0.526 ± 0.0295	-	-	-	-
2-Acetylresorcinol	12.575 ± 0.3048	-	-	-	-
Alkanes					
2-Bromododecane	1.402 ± 0.0448	-	0.721 ± 0.0199	-	1.445 ± 0.0122
Ecosane	1.367 ± 0.0577	-	0.207 ± 0.0532	-	0.188 ± 0.0079
1-Methoxy-2-methylpropane	-	-	2.806 ± 0.1452	-	-
10-Methyl-eicosane	-		-	-	0.974 ± 0.0284

2-Methyl-eicosane	-	-		7.189 ± 5.3044	-
7-Hexyl-eicosane	0.388 ± 0.0353	-	-	-	-
2-Butyl-1, 1, 3-trimethylcyclohexane	-	-	-	0.430 ± 0.0053	0.649 ± 0.0042
Positive 19 alkane	-	-	-	0.106 ± 0.0038	0.446 ± 0.0226
2-Methyl Decane	-	-	-	-	-
Cetadecane	0.253 ± 0.0064	0.199 ± 0.0028	0.207 ± 0.0110	0.467 ± 0.0250	-
2, 6, 10, 14-Tetramethylhexadecane	-	-	0.797 ± 0.0047	-	-
2-Methylcadecane	3.460 ± 0.1645	-	1.757 ± 0.0100	2.246 ± 0.1233	0.997 ± 1.2799
14 alkane	0.599 ± 0.0207	-	0.328 ± 0.0068	-	0.545 ± 0.0279
4-Methyltetradecane	-	-	-	-	0.864 ± 0.6934
2-Methyltetradecane	-	0.638 ± 0.0105	0.541 ± 0.0235	-	-
2-Methyl pentacane	-	-	-	-	2.263 ± 1.7351
Olefins and others					
1 H-benzocyclohepten, 2, 4a, 5, 6, 7, 8, 9 a-octahydro-3, 5, 5-trimethyl-9-methylene-,	0.468 ± 0.1284	0.666 ± 0.0194	0.612 ± 0.1165	0.799 ± 0.0878	0.863 ± 0.1820

Bromate	1.742 ± 0.4866	2.914 ± 0.2000	2.669 ± 0.5006	2.117 ± 1.8436	3.319 ± 0.5882
D-limonene	0.132 ± 0.0037			0.02436	0.254 ± 0.0003
Tetramethyl-pyrazine	10.152 ± 1.1177	10.448 ± 0.2111	10.775 ± 1.4328	9.673 ± 0.4429	17.513± 1.8800
Toluene	1.764 ± 0.0726	-	-	-	-
Cis-(-)-2, 4a, 5, 6, 9a-hexahydrogen- 3, 5, 5, 9-tetramethyl (1H) benzocycloheptene	1.965 ± 0.3465	-	-	3.153 ± 0.1506	-

Note: - Represents no data.

Table S5 index of Alpha diversity of fungi

Sample	Sobs	Chao 1	Shannon	Coverage
D0MH12	43	43	0.550551	1
D7MH12	49	49	0.698943	1
D14MH12	53	53	1.185285	1
D28MH12	15	15	1.012125	1
D45MH12	39	39	1.44422	1

3. Supplemental figure

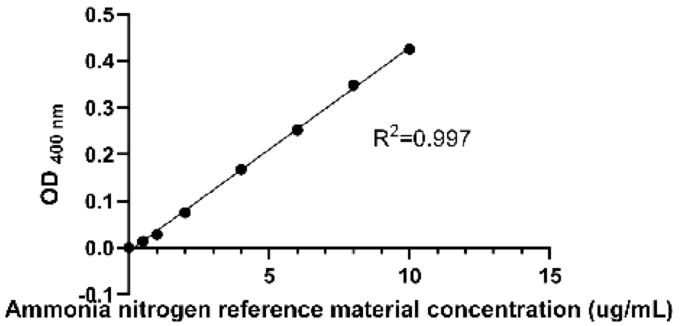

Figure S1. Amino acid nitrogen standard curve.

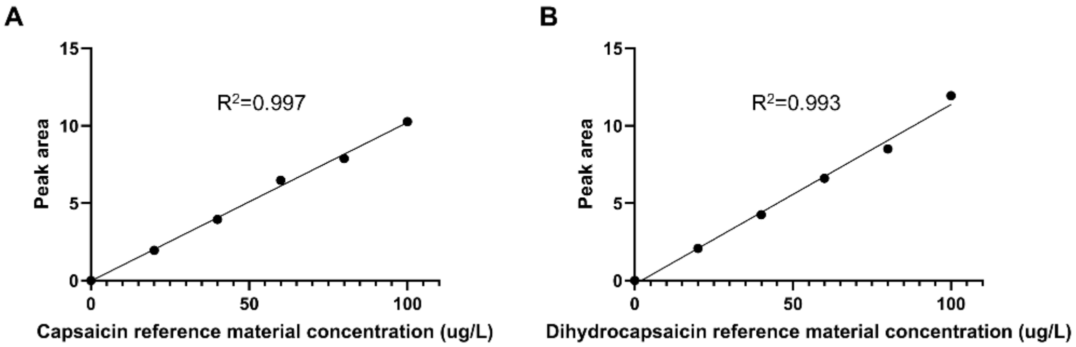

Figure S2. A. Standard curves of capsaicin and B. dihydrocapsaicin.

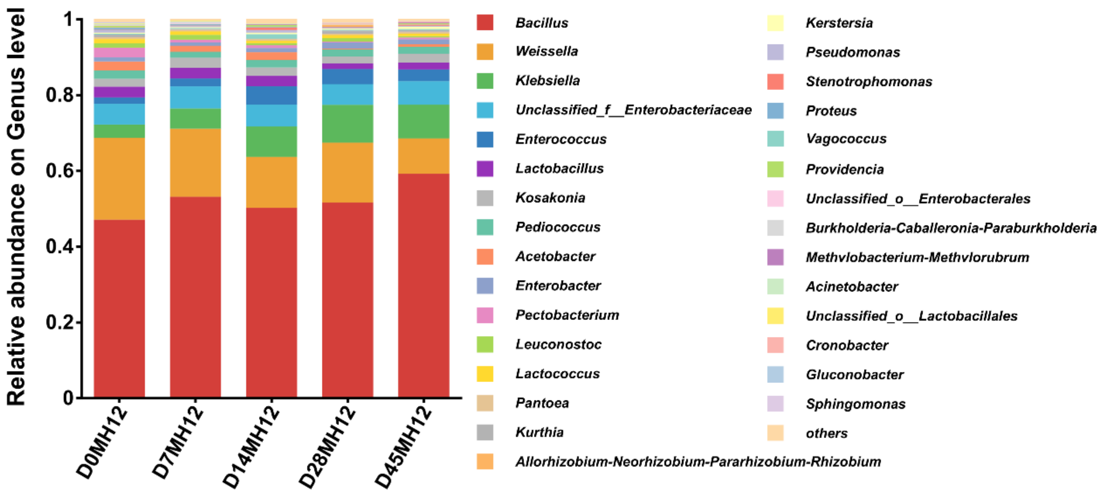

Figure S3. Relative abundances of genus of MH12.

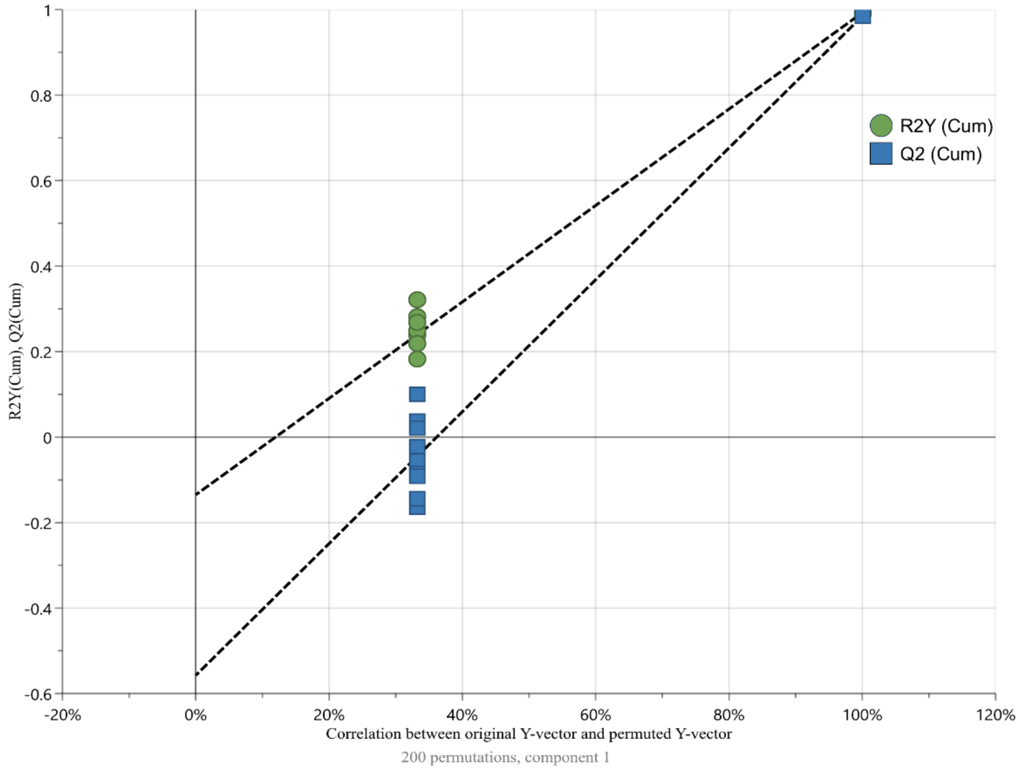

Figure S4. The OPLS-DA models of D0MH12 and D45MH12 in chili mash were fitted with the result plots of 200 permutation.
